# A novel ligand-receptor relationship between families of ribonucleases and receptor tyrosine kinases

**DOI:** 10.1186/s12929-018-0484-7

**Published:** 2018-11-19

**Authors:** Ying-Nai Wang, Heng-Huan Lee, Mien-Chie Hung

**Affiliations:** 1 Department of Molecular and Cellular Oncology, The University of Texas MD Anderson Cancer Center, Unit 108, 1515 Holcombe Boulevard, Houston, TX 77030 USA; 2 Graduate School of Biomedical Sciences, The University of Texas Health Science Center, Houston, TX 77030 USA; 3 Graduate Institute of Biomedical Sciences and Center for Molecular Medicine, China Medical University, Taichung, 404 Taiwan

**Keywords:** Ribonuclease, Angiogenin, Ligand, Serum biomarker, Receptor tyrosine kinase, Epidermal growth factor receptor, Tyrosine kinase inhibitor, Cancer, Targeted therapy

## Abstract

Pancreatic ribonuclease is known to participate in host defense system against pathogens, such as parasites, bacteria, and virus, which results in innate immune response. Nevertheless, its potential impact to host cells remains unclear. Of interest, several ribonucleases do not act as catalytically competent enzymes, suggesting that ribonucleases may be associated with certain intrinsic functions other than their ribonucleolytic activities. Most recently, human pancreatic ribonuclease 5 (hRNase5; also named angiogenin; hereinafter referred to as hRNase5/ANG), which belongs to the human ribonuclease A superfamily, has been demonstrated to function as a ligand of epidermal growth factor receptor (EGFR), a member of the receptor tyrosine kinase family. As a newly identified EGFR ligand, hRNase5/ANG associates with EGFR and stimulates EGFR and the downstream signaling in a catalytic-independent manner. Notably, hRNase5/ANG, whose level in sera of pancreatic cancer patients, serves as a non-invasive serum biomarker to stratify patients for predicting the sensitivity to EGFR-targeted therapy. Here, we describe the hRNase5/ANG-EGFR pair as an example to highlight a ligand-receptor relationship between families of ribonucleases and receptor tyrosine kinases, which are thought as two unrelated protein families associated with distinct biological functions. The notion of serum biomarker-guided EGFR-targeted therapies will also be discussed. Furthering our understanding of this novel ligand-receptor interaction will shed new light on the search of ligands for their cognate receptors, especially those orphan receptors without known ligands, and deepen our knowledge of the fundamental research in membrane receptor biology and the translational application toward the development of precision medicine.

## Background

The family of pancreatic ribonuclease (RNase) is pyrimidine-specific endonuclease found in high quantities in the pancreas of a number of mammals [1]. A bovine version of RNase (bRNaseA), the first enzyme sequenced as a classic model system for protein structure and enzymatic function, is the best-studied member of the family [1, 2]. bRNaseA harbors endoribonuclease enzyme activity at residues His12 and His119, which are critical for the acid base catalysis and stabilizes the transition state via Lys41 by donating a single hydrogen bond to neutralize the excess negative charge on the non-bridging phosphoryl oxygens in the transition state during RNA cleavage [2]. bRNaseA is secreted from β cells in the pancreatic islets of Langerhans and commonly used in research to cleave single-stranded RNAs [2, 3]. The human RNase A (hRNase A) superfamily, which shares sequence and structural similarity with bRNaseA, has 13 known members identified to date that are divided into two subgroups: canonical RNases 1–8 and non-canonical RNases 9–13 [4]. In this review, we will gain more insights into the fifth member of the RNase A superfamily, hRNase5/ANG.

Growth factors and their cognate receptors play essential roles in governing the life cycle of cells and organisms [5, 6]. One of the most extensively studied growth factor receptors is epidermal growth factor receptor (EGFR/ErbB-1), a member of the ErbB receptor tyrosine kinase family long recognized as a primordial type of receptor tyrosine kinase (RTK) that contributes to key steps in both human physiology and diseases [7–9]. Upon extracellular ligand binding, EGFR dimerizes via receptor homodimerization or heterodimerization, leading to subsequent activation of tyrosine kinase activity and downstream cascades of signaling molecules, such as MAPK, PI3K, PLC, and STAT [10, 11]. An EGFR-derived network has been shown to play an oncogenic role in modulating tumor behavior in various cancers such that EGFR has been considered an effective target for anticancer therapies in certain clinical settings [12, 13]. Many monoclonal antibodies (mAbs) occluding the EGFR ligand-binding domain, and small-molecule tyrosine kinase inhibitors (TKIs) targeting EGFR have been approved and are commonly used in the clinic for some cancers [14–16]. Targeted therapy, particularly against oncogenic RTKs, has become one of major cancer therapeutic approaches in recent years [17, 18]. Thus, it is timely and critical to identify biomarkers that can predict response to therapy and therefore be used to stratify patients for effective drug treatment as precision medicine. This review will discuss a newly identified EGFR ligand, hRNase5/ANG, as a serum biomarker for prediction of EGFR-TKI erlotinib response.

### The hRNase A superfamily

The hRNase A superfamily consists of 13 members which genes encoding both canonical RNases 1–8 and noncanonical RNases 9–13 (Table 1). All genes are closely linked on chromosome 14 and encode secretory proteins containing a classical hydrophobic signal peptide at the N-terminus [19–21]. Each RNase maintains an invariant catalytic triad of two histidine (His) residues located at the C-terminus and one lysine (Lys) residue within a consensus CKXXNTF motif, and contains six to eight conserved cysteine residues that form three to four disulfide bonds [1, 22–24]. For instance, the hRNase5/ANG catalytic triad contains active site residues His13, Lys40, and His114 which share sequence similarity with that of bRNaseA containing residues His12, Lys41, and His119. Among all members of the hRNase A superfamily, pancreatic hRNase 1 (hRNase1) and hRNase5/ANG are evolutionarily more related to bRNaseA [25, 26]. hRNase1 is traditionally recognized as the direct ortholog of bRNaseA based on their sequence and structure similarity; hRNase5/ANG is a close homolog of hRNase1 with 35% in sequence identity and 68% homology [25, 26]. However, Raines and colleagues unexpectedly found that the functional bovine homolog of hRNase1 is the bovine brain RNase, not the pancreatic bRNaseA, which support the distinct functions between hRNase1 and bRNaseA [27]. Although the amino acid sequences of RNases 9–13 are only 15–30% identical to the canonical RNase subgroup, several characteristics, including the conserved residues important for folding and structure, and the conserved disulfide bonds suggested that all these proteins share a common ancestry [19]. Intriguingly, hRNase5/ANG perhaps represents the most ancient form of this superfamily because only an RNase 5-like gene but not others has been reported outside the class Mammalia while tracing the origin and evolutionary history [19, 28].

**Table 1:** Summary of characteristics of RNases

RNase	Species	Subgroup	Chromosome	Predicted mass (kDa)^b^	Isoelectric points (pI)	RNase activity^c^
bRNaseA	bovine	–	–	13.7	9.3 [2]	–
hRNase1	human	canonical	14q11.2	17.6	8.6 [22]	0.147
hRNase2/EDN^a^	human	canonical	14q11.2	18.4	8.9 [22]	0.65
hRNase3/ECP^a^	human	canonical	14q11.2	18.4	11.4 [22]	0.048
hRNase4	human	canonical	14q11.2	16.8	8.9 [22]	~hRNase1
hRNase5/ANG^a^	human	canonical	14q11.2	16.6	10.4 [22]	extremely low
hRNase6/k6	human	canonical	14q11.2	17.2	9.1 [22]	0.034
hRNase7	human	canonical	14q11.2	17.4	10.5 [22]	0.021
hRNase8	human	canonical	14q11.2	17.0	8.2 [22]	0.012
hRNase9	human	non-canonical	14q11.2	24.3	n/a	inactive
hRNase10	human	non-canonical	14q11.2	24.0	n/a	inactive
hRNase11	human	non-canonical	14q11.2	22.4	n/a	inactive
hRNase12	human	non-canonical	14q11.2	17.2	n/a	inactive
hRNase13	human	non-canonical	14q11.2	17.8	n/a	inactive

^a^*EDN* eosinophil-derived neurotoxin, *ECP* eosinophil cationic protein, *ANG* angiogenin

^b^Summary of hRNases from Uniprot (www.uniprot.org/uniprot/)

^c^RNase activity was measured against yeast tRNA as per pmol RNA substrate digested/pmol enzyme/sec [22, 36]

All members of the hRNase A superfamily are secretory proteins from various cell types, such as epithelial, endothelial, and immune cells [19]. This superfamily’s role in maintaining antimicrobial activity is generally recognized as part of the host defense system against pathogens, e.g., parasites, bacteria, and virus, and stimulates innate immune response [29–31]. In addition, they possess a number of physiological and biological functions, such as digestion of dietary RNA (hRNase1), angiogenesis (hRNase5/ANG), and antimicrobial host defense (hRNases 2, 3, and 7) [4, 26, 32] (Table 2). A bacterial cell surface receptor for human RNase 7 has been reported to mediate the microbicidal function [33, 34]. Notably, the non-canonical hRNases 9–13 do not possess all of the elements to support ribonucleolytic activity as certain residues essential for their RNase activities are encoded by insertions, deletions, or mutations [4, 19]. Intriguingly, certain RNases, such as hRNase9, do not harbor detectable ribonucleolytic activity but still exhibit antibacterial activity against *E. coli*. [35]. Researchers suggested that the presence of positively charged residues and an amphipathic α-helix conformation enables hRNase9 to permeate the negatively charged cell membrane of micro-organism, leading to the leakage of cytoplasmic components and cell death [35]. Moreover, hRNase5/ANG harbors extremely low enzymatic activity compared with hRNase1 or bRNaseA, and it cleaves standard RNA substrates 10^5^–10^6^ times less efficiently than bRNaseA even though it shares high similarity to hRNase1 in tertiary structure and contains many of the same catalytic residues as bRNaseA [36, 37]. In this section, we will focus on the role of hRNase5/ANG in human diseases, especially on its functions in cancers.

**Table 2:** Summary of tissue specificity and biological process of RNases

RNase	Main expression tissues/cells^a^	Biological processes ^b^
hRNase1	• Pancreas • Gastrointestinal tract • Male tissues • Brain • Appendix • Kidney • Endothelial cells	• Ribonucleolytic activity • Coagulation and inflammation [195]
hRNase2/EDN	• Bone marrow • Spleen • Liver • Lung • Eosinophils • Neutrophils • Monocytes	• Ribonucleolytic activity • Antiviral activity • Chemotactic activity • Neutrophil degranulation
hRNase3/ECP	• Bone marrow • Eosinophils • Neutrophils • Monocytes • T cells	• Ribonucleolytic activity • Antibacterial activity • Antiviral activity • Cytotoxic to mammalian cells • Neutrophil degranulation • Innate immune response
hRNase4	• Liver • Adipose • Colon • Monocytes • B cells /T cells • Endothelial cells	• Ribonucleolytic activity • Protection of neuron degeneration
hRNase5/ANG	• Liver • Spinal cord neurons • T cells • Mast cells • Endothelial cells	• EGFR ligand [46] • Hematopoietic regeneration [81] • Binding to plexin-B2 receptor [101] • Tumorigenesis • Weak ribonucleolytic activity • Neuroprotection • Antibacterial activity • Antifungal activity • Angiogenesis • Ribosomal RNA transcription • Innate immune response
hRNase6/k6	• Bone marrow • Tonsil • Lung • Thymus • Monocytes • Neutrophils	• Ribonucleolytic activity • Antibacterial activity • Antimicrobial activity • Antiviral activity • Innate immune response
hRNase7	• Skin • Tonsil • Female tissues • Gastrointestinal tract • Kidney • Liver • Skeletal muscle • Heart	• Ribonucleolytic activity • Antimicrobial activity • Antibacterial activity • Antifungal activity • Innate immune response • Membrane disruption in other organism
hRNase8	• Placenta • Spleen • Lung • Testis	• Ribonucleolytic activity • Antimicrobial activity • Antibacterial activity • Antifungal activity • Innate immune response
hRNase9	• Male tissues	• Male reproductive functions [4, 196] • Antibacterial activity [35]
hRNase10	• Male tissues • Heart	• Male reproductive functions [4, 196] • Regulation of cell-cell adhesion
hRNase11	• Male tissues	• Male reproductive functions [4, 196]
hRNase12	• Male tissues	• Male reproductive functions [4, 196]
hRNase13	• Male tissues	• Male reproductive functions [4, 196]

^a^ Summary from [196], Uniprot (www.uniprot.org/uniprot/), and The Human Protein Atlas (https://www.proteinatlas.org/)

^b^ Summary from Uniprot (www.uniprot.org/uniprot/)

#### Origin and regulation of hRNase5/ANG

The protein expression of hRNase5/ANG is enhanced during inflammation, pregnancy, and certain pathological conditions, such as cardiovascular disorders [38–41], as well as in several malignant diseases and cancer types, such as pancreatic, colorectal, prostate, ovarian, thyroid, and bladder cancers as well as acute myelogenous leukemia, and multiple myeloma [37, 42–44]. The increased hRNase5/ANG mRNA expression is also found in pancreatic acinar cells and interstitial fibroblasts in the tissues surrounding pancreatic cancer [45]. In pancreatic cancer cells, hRNase5/ANG expression is critically driven by the PTEN/PI3K/Akt activating pathway [46]. Aside from the pancreas, an earlier report indicated that pancreatic-like RNase is detected in serum, urine, and several organs, such as kidney, brain, and spleen [47]. Moreover, hRNase5/ANG can originate from cancer-associated stroma cells, including endothelial cells and fibroblasts [48], which may be upregulated by cytokine stimulation, such as interleukin-6 [49]. Under hypoxic conditions, hRNase5/ANG is upregulated in certain tumor cells [50–52]. Hypoxia can also increase hRNase5/ANG production in dental pulp-derived cells and retinal pigment epithelial cells via an activation of hypoxia-inducible factor-1 signaling [53, 54]. Furthermore, the extent of focal macrophage infiltration is reported to be correlated with the increased hRNase5/ANG expression [55]. On the basis of those previous reports, we speculated that hRNase5/ANG in some patients may be induced by hypoxic stress or proinflammatory cytokines derived from inflammatory cells, e.g., infiltrating macrophages, in the tumor microenvironment. A systematic study in vivo and in vitro would be required to further understand the reasoning for ANG production.

#### Role of hRNase5/ANG in angiogenesis

It is well recognized that hRNase5/ANG induces blood vessel formation in vitro and in vivo to promote angiogenesis; hence, hRNase5 is also named angiogenin/ANG [56–58]. Intriguingly, the limited ribonucleolytic activity of hRNase5/ANG is reported to be essential for its angiogenic effect because site-directed mutagenesis of its active site residues His13 and His114 abolished its ribonucleolytic activity and inhibited hRNase5/ANG-mediated angiogenesis [59]. In addition, hRNase5/ANG binds to membrane actin to accelerate plasminogen activation [60], increases endothelial cell migration and proliferation by transmitting signals into the cytoplasm, such as PLC, Akt, and ERK, through an association with a putative 170-kDa cell surface protein [61–64], and translocates into the nucleus in endothelial cells to enhance ribosomal RNA transcription [65]. Furthermore, Hu and colleagues demonstrated that hRNase5/ANG also enters the nucleus of cancer cells and induces ribosomal RNA transcription and the corresponding cell proliferation [66].

#### Role of hRNase5/ANG in cancers

**Fig. 1:**
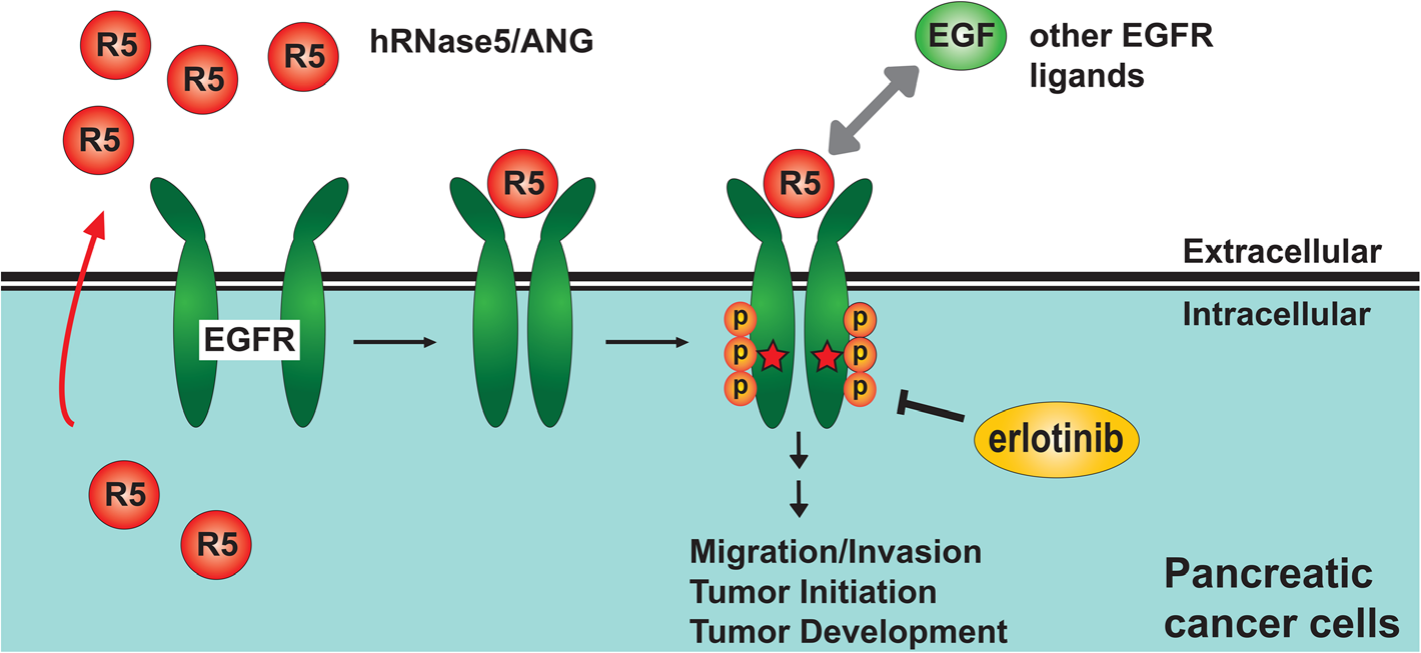
A diagram of hRNase5/ANG as an EGFR ligand and a serum biomarker for prediction of erlotinib sensitivity in pancreatic cancer. Secretory hRNase5/ANG acting as an EGFR ligand associates with extracellular domain of EGFR, which in turn induces EGFR dimerization and phosphorylation/activation (red stars), leading to tumorigenesis and increased sensitivity to erlotinib treatment in pancreatic cancer patients. In addition, hRNase5/ANG also competes with other EGFR ligands, such as EGF, for EGFR binding, due to the partially overlapped epitope of EGFR binding region between hRNase5/ANG and EGF. The scale of the diagram does not reflect the relative sizes of different molecules

Because RNase activities are elevated in several types of malignancies, they were proposed as a diagnostic biomarker over three decades ago [37, 67–70]. Nevertheless, it had not been further developed into clinical practice largely due to the insufficient sensitivity and specificity from a lack of appropriate methods to measure specific RNase activity [71]. Increased levels of hRNase5/ANG were reported to correlate with pancreatic cancer occurrence and aggressiveness [72]. Most recently, Hung and colleagues identified hRNase5/ANG as an EGFR ligand to induce its binding to EGFR and activate EGFR signaling in an RNase catalytic-independent manner, which highlights an oncogenic role of the hRNase5/ANG-EGFR axis in pancreatic cancer [46]. A high hRNase5/ANG level, which in turn increases EGFR TKI erlotinib sensitivity, was further demonstrated to serve as a serum biomarker to predict erlotinib response in pancreatic ductal adenocarcinoma patients [46] (Fig. 1). In prostate cancer, hRNase5/ANG was also identified as an oncogene, as shown by its role in angiogenesis, a molecular target for the treatment, and a potential diagnostic biomarker [73–75]. Moreover, hRNase5/ANG plays a role in epithelial-mesenchymal transition (EMT) in squamous cell lung carcinoma to promote cell proliferation, migration, and invasion capacity through direct upregulation of a non-histone chromosomal high-mobility group protein [76, 77]. In addition, hRNase5/ANG functionally interacts with the transcription-activation domain 2 of p53 tumor suppressor and inhibits p53 functions to mediate anti-apoptosis and survival of cancer cells [78, 79]. A synthetic compound, terrain, was demonstrated to inhibit tumor cell proliferation in head and neck cancer by suppressing hRNase5/ANG production [80]. Researchers further showed that hRNase5/ANG promotes hematopoietic regeneration by simultaneously enhancing stem cell quiescence and myeloid-restricted progenitor cell proliferation [81]. Of note, the cellular uptake of hRNase5/ANG requires multiple pathways, including micropinocytosis, similar to the mechanism of bRNaseA endocytosis, and is largely clathrin/dynamin independent [82, 83]. Cytoplasmic hRNase5/ANG is tightly associated with ribonuclease/angiogenin inhibitor 1 (RNH1) which prevents hRNase5/ANG from random cleavage of cellular RNA; however, hRNase5/ANG can evade RNH1 by translocation into the nucleus via protein kinase C- and cyclin-dependent kinase-mediated phosphorylation [84, 85]. Altogether, the evidence from various cancer types supports a positive regulation of hRNase5/ANG in tumorigenesis.

#### Role of hRNase5/ANG in neurodegenerative diseases

In addition to angiogenic and oncogenic roles, hRNase5/ANG also acts as a neurotrophic/neuroprotective factor because it has been reported to play a role in neovascularization by protecting motor neurons from hypoxia and stimulating neurite outgrowth and pathfinding, and is highly expressed in the human spinal cord [86–89]. In 2004, hRNase5/ANG was identified for the first time as one of the key genes associated with amyotrophic lateral sclerosis (ALS) [90], a common neurodegenerative disease that affects nerve cells in the brain and the spinal cord [91, 92]. The loss-of-function mutations in hRNase5/ANG have been implicated in patients with ALS [93–96]. Accordingly, enhancing hRNase5/ANG expression or its activities have shown potentially therapeutic benefits for ALS patients [97, 98]. In addition, some ALS patients harboring ANG variants also showed signs of Parkinson disease, presenting a genetic link of hRNase5/ANG between ALS and Parkinson disease [99, 100]. Notably, a recent report indicated that plexin-B2 (PLXNB2) is the functional receptor of hRNase5/ANG in several cell types, including endothelial, cancer, neuronal, and normal and malignant stem/progenitor cells [101]. Disrupting the interaction between hRNase5/ANG and PLXNB2 by competition using a PLXNB2 monoclonal antibody dampened hRNase5/ANG-mediated physiological and pathological functions, suggesting that targeting PLXNB2 may have therapeutic potential by modulating hRNase5/ANG activities [101].

### RTK family

RTK family, the most widely recognized family of enzyme-linked cell surface receptors, plays fundamental roles in a wide spectrum of cellular processes, such as cell proliferation, migration, differentiation, and survival. There are 58 known human members of RTK family divided into 20 classes [102, 103]. A prototypical RTK, which contains an extracellular ligand-binding domain (ECD), a transmembrane helix, and a cytoplasmic domain comprising a tyrosine kinase domain and a carboxyl terminal domain, mediates signal transduction by binding to extracellular ligands consisting of different variety of growth factors [104]. Dysregulation of the RTK members or these ligand-receptor axes are found in many pathological situations including cancers [105–107]. While therapeutics targeting RTKs and their downstream molecules have shown anti-cancer efficacy in the clinic, acquired drug resistance invariably occurs [108]. Thus, understanding the RTK signaling will significantly contribute to the potential of developing more comprehensive strategies for clinical application. In this section, we will describe the well-studied EGFR as a model RTK and its signaling modes, ligands, and therapeutic roles in human cancers.

#### EGFR canonical signaling mode

EGFR plays a fundamental role in both physiological and pathological settings, which serves as a model for investigating the signaling and functions of other cell surface RTKs. The canonical view of signal propagation via the transmembrane EGFR is well documented such that EGFR located at the cell surface, following the initial interaction with extracellular growth factors/ligands, serves as a mediator of intracellular signaling cascades [109–111]. In response to ligand binding, EGFR drives conformational changes to form homo- or hetero-oligomers and triggers autophosphorylation on its intracellular tyrosine kinase domain, leading to subsequent tyrosine kinase activation. The activated EGFR is internalized into cytoplasmic vesicles via clathrin-dependent and -independent endocytosis, succeeded by fusion with the early endosomes en route to the lysosomes for degradation or back to the cell surface [112, 113]. The phosphorylated residues of EGFR function as docking sites for adapter proteins such as Grb2 in the cytoplasm to recruit substrates to be phosphorylated to transmit downstream signal transduction involving pathways, such as Ras-Raf-MEK-ERK, PI3K-AKT, PLCγ-PKC, and JAK-STAT, to mediate the corresponding cellular processes upon ligand stimulation [10, 11]. Evolutionarily, the canonical downstream Ras-ERK pathway of RTK is highly conserved from *C. elegans* to humans [114]. Recently, Liang et al. surprisingly found that in the absence of EGF, a phosphorylated EGFR dimer loaded with core signaling adapters as prepared by a chemical-genetic strategy was not sufficient to activate Ras-MAPK pathway, suggesting that the binding of EGFR ligands, such as EGF, contributes to conformational changes which is necessary for higher-order oligomerization and efficient signal transduction [115].

#### EGFR non-canonical signaling mode

In addition to the canonical signaling mode, there is increasing evidence to show the unique transportation of EGFR that accompanies its associated biological functions in which the internalized EGFR can be shuttled from the cell surface to different subcellular compartments such as the mitochondria and the nucleus [116–118]. For instance, upon EGF stimulation, full-length EGFR binds to the mitochondrial protein cytochrome c oxidase subunit II to regulate apoptosis [119, 120]. The mitochondria-located EGFR may play a role in autophagy which is involved in the production of ATP and reactive oxygen species regulated by mitochondrial dynamics [121]. Moreover, mitochondrial accumulation of EGFR induces mitochondrial fission through inhibition of mitofusin-1 protein and promotes cell migration and invasion in lung cancer [122]. A later study indicated that in lung cancer, Tid-1-S, a short form of Tid1 also known as mitochondrial heat shock protein 40 (mtHSP40), interacts with EGFR in the cytosol and governs EGFR translocation into the mitochondria, where an EGFR/Tid1-S/mtHSP70 complex enhances cell metastasis and tumor progression [123]. Non-canonical localization of cell surface RTKs in the nucleus, termed membrane receptors in the nucleus (MRIN) [124], has been demonstrated for 11 of the 20 RTK classes [125, 126]. Nuclear functions of EGFR, which is one of the best-documented RTKs in the MRIN field, include transcriptional co-activator, protein kinase, and protein-protein interactor, which are responsible for various cellular responses, such as cell proliferation, DNA replication, DNA damage repair, and resistance to certain anticancer therapies, including DNA damage events (irradiation and cisplatin) and EGFR-targeted drugs (cetuximab and gefitinib) [127–129]. For example, Dittmann and colleagues found that nuclear EGFR renders cells resistant to irradiation by binding and stabilizing mRNA involved in the Warburg effect and triggering a metabolic switch to increase lactate production, which is associated with increased therapeutic resistance [130]. They further identified a new role for nuclear EGFR upon exposure to irradiation in modulating the stability and translation of mRNAs associated with HIF1a/VEGF signaling in a miRNA-directed manner [131]. Moreover, Wheeler and colleagues found a positive cooperation between nuclear EGFR and Src family kinases (SFKs) activity in acquired resistance to EGFR mAb cetuximab [132]. Recently, another RTK called AXL was clarified to trigger the nuclear accumulation of EGFR by increasing the expression of two SFK members and an EGFR family ligand neuregulin-1, which may contribute to the resistance to cetuximab that targets the extracellular domain of EGFR [133]. In addition, a series of studies by Hung and colleagues showed that multiple nuclear proteins, including PCNA (proliferating cell nuclear antigen), histone H4, and ATM (ataxia telangiectasia mutated), can be phosphorylated by EGFR in a specific tyrosine site, which affects their functions in mediating DNA replication, DNA synthesis and repair, and DNA damage response [134–136]. The authors further demonstrated that EGFR can physically associate with a DNA binding protein RNA helicase A and certain transcriptional factors, such as STAT3 (signal transducers and activators of transcription 3), to regulate target gene transcription [137, 138]. Collectively, a better understanding of the non-canonical transport of EGFR may shed light on the cell surface receptor biology and potential therapeutic implications.

#### EGFR ligands

To date, EGFR is known to be activated by the binding of various ligands including EGF, betacellulin (BTC), heparin-binding EGF-like growth factor (HB-EGF), transforming growth factor-α (TGFα), amphiregulin (AREG), epiregulin (EREG), epigen (EPGN), and a recently identified hRNase5/ANG [46, 139–142] (Table 3). Based on the binding affinity with EGFR, these ligands fall into two groups as follows: high-affinity EGFR ligands (EGF, BTC, HB-EGF, TGFα, and hRNase5/ANG) bind with a dissociation constant (Kd) between 1 and 100 nM and low-affinity EGFR ligands (AREG, EREG, murine EREG, EPGN, and bRNaseA) bind with a Kd greater than 100 nM [46, 143–145]. The lower binding affinity of bRNaseA to EGFR, compared with that of hRNase5/ANG to EGFR, may be a result of its non-human origin [46]. After ligand binding, the activated EGFR triggers a complex signaling process, resulting in the transmission of intracellular trafficking events and leading to EGFR-dependent cellular responses, such as cell proliferation, differentiation, and motility [110, 146]. Individual EGFR ligands can induce distinct downstream signals qualitatively and quantitatively, and thus promoting different effects on cellular responses via stabilization of EGFR dimers with distinct structures [147]. Intriguingly, certain EGFR ligands, such as EGF, pro-TGF-α, pro-HB-EGF, and hRNase5/ANG have been detected in the nucleus [66, 148–150]. Together with the knowledge of nuclear localization of EGFR, the association between EGFR and its ligands may also occur in the nucleus and contribute to certain biological functions. Indeed, a cross-linking experiment between EGF and EGFR has been demonstrated the EGF-EGFR complex in the nucleus [151]. More studies are required to dissect the functions of these ligand-receptor pairs in the nucleus.

**Table 3:** Summary of EGFR ligands

EGFR ligand	Predicted mass (kDa)	Binding affinity (Kd)	Group	Reference
EGF^a^	6.2	0.6 nM; 1.8 nM	high	[46, 143, 145]
BTC	9.8	1.4 nM	high	[143, 145]
HB-EGF	9.7	7.1 nM	high	[143, 145]
TGFα	5.6	9.2 nM	high	[143, 145]
hRNase5/ANG	16.6	41.6 nM	high	[46]
AREG^a^	11	217.4 nM; 350 nM	low	[46, 145]
EREG	5.6	100-fold lower than EGF	low	[143, 197]
EREG^b^	5.5	2.8 μM	low	[145]
EPGN	7.9	> 500 nM	low	[145]
bRNaseA^c^	13.7	885.3 nM	low	[46]

^a^Kd values from two independent reports

^b^murine epiregulin

^c^bovine RNase A

#### EGFR as a therapeutic target in cancers

As a well-known oncogene that is critical to tumorigenesis, EGFR has been evaluated in clinical trials as the target of effective cancer therapies by mAbs (e.g., cetuximab and panitumumab) or TKIs (e.g., erlotinib, gefitinib, afatinib, and osimertinib) [14–16, 152]. For instance, treatment with cetuximab has resulted in improved overall patient survival in colorectal cancer and head and neck cancer [153, 154]. In contrast, EGFR TKIs have been approved to treat both lung and pancreatic cancers [155–157]. In the case of lung cancer, it is well known that EGFR-activating mutations can be used as a biomarker to stratify patients for EGFR TKI treatment as lung cancer harboring EGFR mutations is addicted to EGFR activation and thus sensitive to EGFR TKIs. This biomarker-guided treatment has a prolonged lifespan of a number of lung cancer patients [157]. However, such mutations are infrequent in pancreatic cancer patients [158, 159]. Although erlotinib has resulted in a therapeutic advantage in patients with advanced pancreatic cancer [156], it has only provided marginal clinical benefit in general without any predictive biomarkers [160–162]. Analogous to the EGFR-activating mutations in lung cancer, identifying such addiction to the EGFR pathway in pancreatic cancer could lead to improved response to EGFR TKI treatment for selective pancreatic cancer patients. It is worthy to note that another EGFR-directed clinical trial testing cetuximab as adjuvant therapy with gemcitabine in patients with advanced pancreatic adenocarcinoma did not improve the overall survival compared with patients treated with gemcitabine alone [163, 164]. The limited benefit of EGFR-targeted therapies in pancreatic cancer calls for identification of potential biomarkers by stratifying patients to predict drug response. A new and timely report (see next section) provides insights into the potential of hRNase5/ANG serving as a non-invasive serum biomarker to stratify pancreatic cancer patients for erlotinib therapy [46].

### A novel ligand-receptor relationship: hRNase5/ANG vs. EGFR

Hung and colleagues recently demonstrated that hRNase5/ANG functions as a ligand of EGFR and a serum biomarker to predict EGFR-TKI erlotinib response in patients with pancreatic adenocarcinoma [46]. The newly identified role of hRNase5/ANG furthers our understanding of EGFR in basic research for the ligand-receptor cognate signaling and translational application for the targeted therapeutics.

**Fig. 2:**
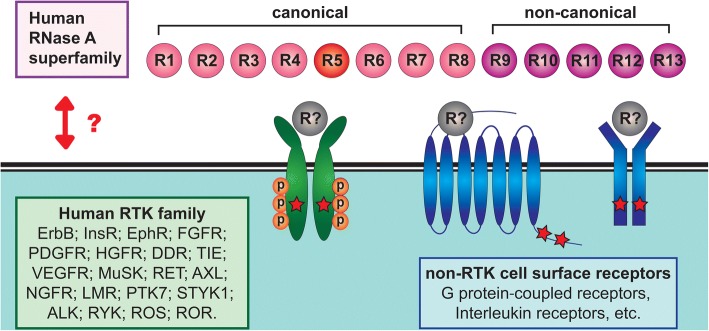
A proposed model of the ligand-receptor cognate signaling through a ligand-like function of RNases. The human RNase A superfamily contains 13 known members that are divided into canonical (RNases 1–8) and non-canonical (RNases 9–13) subgroups. The fifth member of the RNase A superfamily, hRNase5/ANG, functions as an EGFR ligand. Identification of the hRNase5/ANG-EGFR axis raises an interesting question of whether other RNase family members may play a ligand-like function, linking the two unrelated protein families, namely RNases and RTKs or non-RTK cell surface receptors. Red stars indicate receptor activation. The scale of the diagram does not reflect the relative sizes of different molecules

RNase is a secretory enzyme that normally exerts its endoribonuclease activity to degrade RNAs and is critical for host defense to be set against pathogens [2, 30]. Wang and Lee et al. [46] initially designed a pilot test utilizing bRNaseA treatment to broadly remove RNAs in studying potential biological processes of extracellular RNAs, which are known to act as signaling molecules in cell-to-cell communication and serve as disease biomarkers [165, 166]. Unexpectedly, they found that bRNaseA can promote epithelial-mesenchymal transition-like morphological changes and oncogenic signaling in multiple cancer cell lines. Because the process of oncogenesis is tightly associated with the activation of tyrosine kinase cascades [167, 168], the authors performed a human antibody array for phospho-RTKs and identified EGFR RTK as the dominant cell surface target activated by bRNaseA. The authors further demonstrated the association between EGFR and bRNaseA in conveying EGFR downstream signaling, supporting that bRNaseA functions as an EGFR ligand. The human counterpart of bRNaseA, hRNase5/ANG, also exhibits similar ligand-like function in vitro and in vivo. Notably, the RNase enzymatic activity of bRNaseA and hRNase5/ANG is not required for EGFR binding or activation. This catalytic-independent function of RNases raises an interesting question of whether other catalytic-deficient RNases, such as the non-canonical RNases 9–13 [4, 19], may also play non-canonical roles as a cognate ligand of RTK (Fig. 2). Further systematic study is required to unveil the potential ligand-like roles of other RNases.

In the general context of tumorigenesis mediate by EGFR, the NIH-3 T3 fibroblast cell line, which lacks endogenous EGFR, is a well-established system to validate its cognate ligands such as EGF, in which both ectopic expression of EGFR and EGF are required for the enhanced transformed phenotypes [169]. Similarly, hRNase5/ANG has been demonstrated in NIH-3 T3 stable clones as a bona fide EGFR ligand to promote tumorigenesis in vivo, which requires EGFR kinase activity [46]. A comparison between EGF- and hRNase5/ANG-treated cells indicated a high similarity in transcriptome changes by RNA deep sequencing, suggesting that hRNase5/ANG modulates gene transcription by inducing signaling events similar to EGF. Additionally, hRNase5/ANG belongs to the class of high-affinity EGFR ligands with a Kd value between 1 and 100 nM that is similar to EGF [145] (Table 3). The binding of hRNase5/ANG to EGFR also requires domains I and III of EGFR-ECD, known to bind EGF [170, 171] such that the binding epitope partially overlaps with the EGFR binding region to EGF. It would be worthwhile to be further determining whether a direct contact between hRNase5/ANG and EGFR exists by structural analysis.

### hRNase5/ANG as a serum biomarker for EGFR-targeted therapy

EGFR is a well-known oncogene and an effective rational target of anti-cancer therapies. Of note, EGFR and its downstream signaling are required for initiation of *KRAS*-driven pancreatic tumorigenesis [172, 173], one of the most lethal human malignancies in the past decades [174]. Of note, frequency of EGFR overexpression is about 30 to 95% in pancreatic cancer [175]. Several lines of evidence show that EGFR and its activation are positively correlated with liver metastasis and pancreatic metaplasia [176–179]. Collectively, these findings suggested that EGFR is closely associated with tumor initiation, development, and metastasis in pancreatic cancer. Unlike EGFR mAb cetuximab [163], even though EGFR TKI erlotinib is approved to treat pancreatic cancer, negligible improvement in a group of responsive population was observed [156]. Thus, pancreatic cancer continues to be a disease without effective therapeutics and identification of predictive biomarkers for the subpopulation of pancreatic cancer patients who may be more likely to respond to erlotinib will be important and beneficial to those responders [160–162].

Previously, elevated serum level of hRNase5/ANG was shown to correlate with poorer patient survival in pancreatic cancer [72]. In line with those findings, Wang and Lee et al. reported that plasma hRNase5/ANG, but not other two EGFR traditional ligands, EGF and TGF-α, was significantly elevated in pancreatic cancer patients [46]. Moreover, a positive correlation between hRNase5/ANG and EGFR activation was observed in human pancreatic tissue microarrays, supporting the pathological relevance of the hRNase5/ANG-EGFR relationship in pancreatic cancer. In addition, increased expression of murine RNase5 and EGFR activation are associated with tumor development in the *Kras*^*G12D*^-driven transgenic pancreatic cancer mouse model [46, 180]. An oncogenic role of hRNase5/ANG in pancreatic cancer through activation EGFR phosphorylation rendered cancer cells more addicted to the EGFR pathway and sensitive to EGFR TKI erlotinib treatment in vitro and in vivo [46]. This oncogene addiction effect was validated in a cohort of pancreatic cancer patients by retrospective studies such that patients with high concentrations of plasma hRNase5/ANG responded well to erlotinib treatment, implying that hRNase5/ANG has potential to serve as a serum biomarker to predict erlotinib response and select pancreatic cancer patients who would be more responsive to EGFR-targeted therapy [46]. It would be worthwhile to further evaluate hRNase5/ANG as a predictive biomarker for EGFR inhibitor therapy in pancreatic cancer in a systematic way through prospective clinical trials in the future.

### Future perspective

This newly discovered hRNase5/ANG-EGFR ligand-receptor cognate signaling pair points to several directions worthwhile to be further pursued, including at least the following: First, the identification of the hRNase5/ANG-EGFR axis supports the notion that the RNase family members may play the ligand-like function to bridge two unrelated protein families, which provides a new direction toward the search for RNase ligands and their cognate receptors, including RTKs and/or cell surface receptors other than RTKs (Fig. 2). In particular, there are several so-called orphan receptors whose activating ligands have yet to be identified, such as ErbB-2, ROS proto-oncogene 1, Ror1 (receptor tyrosine kinase-like orphan receptor 1), Ryk (related to receptor tyrosine kinase), a number of G protein-coupled receptors, interleukin-1 receptor 9, and interleukin-1 receptor 10 [8, 181–184]. Among them, Ror1 and Ryk have been reported to bind to a Wnt protein, but the molecular mechanisms through which these receptors transmit the Wnt signaling remain poorly defined [181, 185]. Of note, another RTK namely ALK (anaplastic lymphoma kinase) had been considered as an orphan receptor with no endogenous ligands until recently when a cytokine termed ALKAL (ALK and LTK ligand; also termed FAM150 or augmentor) was proposed as an in vivo ligand of the ALK family of RTKs in human neuroblastoma cells [186–188]. A more systematic study should be carried out to explore the potential ligand-like roles of other RNases, which could have potentially significant impact on receptor biology in basic science.

**Fig. 3:**
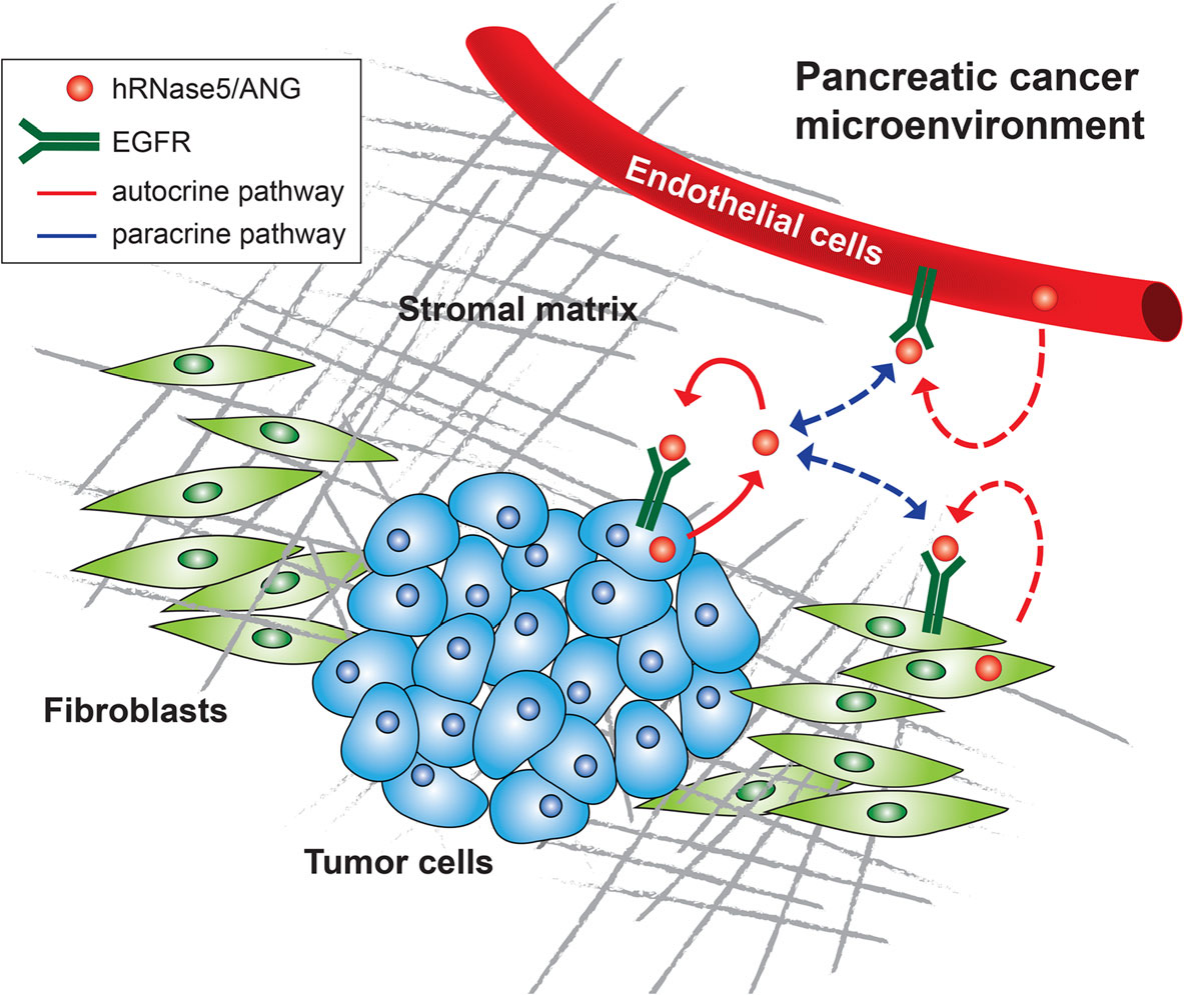
A proposed model of the interplay between hRNase5/ANG and EGFR in modulating the pancreatic tumor microenvironment via an autocrine or paracrine pathway. Secretory hRNase5/ANG can originate from pancreatic tumor cells and bind to EGFR on the tumor cell surface in an autocrine manner. hRNase5/ANG can also be secreted from stroma matrix, including tumor-associated endothelial cells and tumor-associated fibroblasts, where it may bind to EGFR on the cell surface of endothelial cells and fibroblasts, respectively, and trigger EGFR signaling to form autocrine stimulation. In contrast, hRNase5/ANG derived from endothelial cells or fibroblasts may associate with cell surface EGFR of tumor cells, which may stimulate tumor-associated EGFR signaling in a paracrine manner. hRNase5/ANG secreted from tumor cells may interact with EGFR on endothelial cells or fibroblasts to play a role in angiogenesis or other processes. The scale of the diagram does not reflect the relative sizes of different molecules

Second, whether the interplay between hRNase5/ANG and EGFR identified in pancreatic cancer also contributes to the signaling modulation in the pancreatic tumor microenvironment where a dense stromal matrix, including endothelial cells and fibroblasts, is a prominent histopathological hallmark [189]. Since EGFR is shown to be highly expressed in tumor-associated endothelial cells, and both secreted and cellular hRNase5/ANG can be detected in endothelial cells [48, 190], it is possible that, similar to the findings in pancreatic cancer, secretory hRNase5/ANG from tumor-associated endothelial cells may bind to EGFR on the endothelial cell surface, leading to the activation of EGFR signaling in an autocrine manner. Similar autocrine stimulation mediated by the hRNase5/ANG-EGFR pair also has potential to occur in the tumor-associated fibroblasts, where both proteins have considerable amounts of expression [191, 192]. On the other hand, hRNase5/ANG originating from endothelial cells or fibroblasts may interact with EGFR on the cell surface of tumor cells which may result in oncogenic transformation via a paracrine pathway. Likewise, hRNase5/ANG secreted from cancer cells may associate with EGFR on the endothelial cell to play a role in angiogenesis. It would be of interest to further address the regulatory mechanisms and functions of hRNase5/ANG and EGFR, either autocrine or paracrine in different cells in the pancreatic tumor microenvironment (Fig. 3).

Finally, considering hRNase5/ANG as a predictive biomarker in pancreatic cancer, a subset of patients with hRNase5/ANG^high^-EGFR^+^ population (around 30% based on plasma hRNase5/ANG estimation [46]) may benefit from erlotinib treatment. Besides pancreatic cancer, whether this oncogenic addiction through activation of the hRNase5/ANG-EGFR axis exists in other tumor types remains to be determined. If so, whether hRNase5/ANG generally serves as a serum biomarker to predict response to EGFR-targeted therapies in such malignancies is important and worthwhile to be further investigated. For instance, high concentrations of serum hRNase5/ANG have been observed in patients who suffer from various cancer types, such as colorectal cancer, lung cancer, and acute myelogenous leukemia [44, 55, 193], in which EGFR has been well studied to be a therapeutic target in clinical practice [154, 157, 194]. It would be of interest to further determine whether hRNase5/ANG can be used as a predictive biomarker for EGFR-targeted therapies in those with hRNase5/ANG^high^-EGFR^+^ tumors.

## Conclusions

Protein families of RNases and RTKs are considered two unrelated families associated with distinct biological functions. In this review, we illustrated a novel ligand-receptor relationship between RNase and RTK families with the hRNase5/ANG-EGFR pair by exhibiting an intrinsic role of hRNase5/ANG as a unique ligand of EGFR RTK [46]. Binding of hRNase5/ANG to EGFR triggers oncogenic transformation independently of the RNase’s catalytic activity. Moreover, high plasma hRNase5/ANG level of pancreatic cancer patients can predict better treatment response to erlotinib with the potential to serve as a serum biomarker to stratify patients for erlotinib treatment [46]. Investigating the relationship between RNase and RTK families systematically may shed new light on our knowledge of ligand-receptor biology. As many of RTKs are therapeutic targets, this recently identified RNase-RTK ligand-receptor pair may open a new avenue in biomarker-guided treatment options.

## Abbreviations

ALK: Anaplastic lymphoma kinase
ALKAL: ALK and LTK ligand
ALS: Amyotrophic lateral sclerosis
ANG: Angiogenin
AREG: Amphiregulin
bRNaseA: Bovine ribonuclease A
BTC: Betacellulin
ECD: Extracellular ligand-binding domain
EGF: Epidermal growth factor
EGFR: Epidermal growth factor receptor
EMT: Epithelial-mesenchymal transition
EPGN: Epigen
EREG: Epiregulin
HB-EGF: Heparin-binding EGF-like growth factor
hRNase1: Human ribonuclease 1
hRNase5: Human ribonuclease 5
Kd: Dissociation constant
mAbs: Monoclonal antibodies
MRIN: Membrane receptors in the nucleus
mtHSP: Heat shock protein
PLXNB2: Plexin-B2
RNase: Ribonuclease
RNH1: Ribonuclease/angiogenin inhibitor 1
Ror1: Receptor tyrosine kinase-like orphan receptor 1
RTKs: Receptor tyrosine kinases
Ryk: Related to receptor tyrosine kinase
SFKs: Src family kinases
TGFα: Transforming growth factor-α
TKIs: Tyrosine-kinase inhibitors
